# Does county financial marketization promote high-quality development of agricultural economy?: Analysis of the mechanism of county urbanization

**DOI:** 10.1371/journal.pone.0298594

**Published:** 2024-03-06

**Authors:** Yang Liu, JunFu Cui, Liang Feng, Hua Yan

**Affiliations:** 1 School of Economy, Shandong Women’s University, Jinan, China; 2 College of Economics and Management, Northeast Agricultural University, Harbin, China; National University of Sciences and Technology, PAKISTAN

## Abstract

China’s agricultural economy has been hindered by insufficient accumulation of agricultural capital and credit constraints. It is worth investigating whether China’s county financial marketization reform policy can alleviate these constraints and promote high-quality development of the agricultural economy (HQDAE). This paper presents an empirical analysis of the impact of county financial marketization reform on the HQDAE, based on county panel data. The focus is on the mechanism of county urbanization in the above relationship. The results show that county financial marketization has a significant non-linear impact on the HQDAE. Specifically, it has a ’U-shaped’ impact on the overall growth of the agricultural economy and an inverted ’N-shaped’ impact on the quality improvement of the agricultural economy. Secondly, the relationship between county financial marketization and the HQDAE is influenced by a threshold effect based on the level of county urbanization. As the level of county urbanization increases, the promoting effect of county financial marketization on HQDAE also increases significantly. Additionally, county financial marketization helps to promote county urbanization and accelerate urban-rural integration, which in turn leads to HQDAE. The research in this paper suggests that county-level local governments should promote a differentiated county financial system. In the early stages of financial market-oriented reform, the government should enhance the capacity of financial services in rural areas through tax breaks, policy incentives, and other measures to prevent financial leakage from agriculture. In the later stages of financial marketization reform, the government should strengthen financial supervision to prevent financial resources from being diverted from industry to capital. Moreover, to achieve the HQDAE, it is necessary to promote county financial market-oriented reform and accelerate the construction of county urbanization. This will help break the dual economic structure of urban and rural areas and promote the flow of financial capital, technology, and human capital from county cities to rural areas.

## 1 Introduction

According to the Sustainable Development Goals Report, the number of people suffering from hunger in the world reached 780 million in 2022, and the number of people living in extreme poverty increased by 93 million worldwide. Promoting agricultural economic development to eradicate hunger and poverty has become the most important agenda for global sustainable development [1]. Over the past 40 years of reform and opening up, China’s agricultural economy has made significant achievements in effectively solving the problem of insufficient food supply. Additionally, China has made significant contributions to global poverty reduction by comprehensively eradicating absolute poverty [2]. However, China’s agricultural sector faces significant challenges, including high input costs, low efficiency, and serious land pollution, which have resulted in poor competitiveness when compared to the urban industrial economy [3]. The goal of High-quality development of agricultural economy (HQDAE) is to achieve sustainable development of the agricultural economy by improving total factor productivity and promoting agricultural modernization while ensuring national food security, promoting farmers’ income growth, and improving the ecological environment [4,5]. Therefore, finding a sustainable and efficient way to promote HQDAE and achieve the goal of sustainable development with zero hunger and poverty is an urgent issue that needs to be addressed.

Finance plays a crucial role in the modern economy, serving functions such as resource allocation and risk management. Financial support is essential for promoting HQDAE [6]. However, China’s agricultural development has long faced issues such as inadequate accumulation of agricultural capital and an imbalance in the allocation of urban and rural resource factors [7]. Furthermore, the financial control issues that previously existed in rural areas of China have resulted in ineffective allocation of rural finances and increased constraints on agricultural credit [6]. To enhance the rural financial service system and decrease government control over the rural financial market, China has implemented reforms to liberalize rural financial institutions and marketize interest rates since 2006 [8]. This has improved the efficiency of rural financial allocation through market-based competition, providing financial resources for agricultural development [9]. According to statistics, by the end of 2020, the number of bank branches in China’s rural areas had reached 23.76 million. China has gradually formed a diversified system of financial institutions, including large state-owned commercial banks, rural commercial banks (including rural credit cooperatives), village banks, and other banks. In October 2015, China’s central bank completely abolished interest rate control, resulting in a market-oriented deposit and loan interest rate system. Can the financial marketization reform in the county alleviate agricultural credit constraints and promote the HQDAE? This question deserves further study.

Research on the impact of finance on the agricultural economy is a current topic in the field of economic and rural finance. The relevant research presents three theoretical disputes: ’inhibition theory’, ’promotion theory’, and ’nonlinear theory’ [10]. Due to the high risk and low return nature of the agricultural sector in China, financial institutions tend to avoid it. According to [11], full liberalization of China’s county finances and market allocation of financial resources may result in an excessive flow of financial resources to the industrial sector. However, the government’s ongoing efforts to reform the county financial market will improve the effectiveness of financial resource allocation and intensify competition among financial institutions [12]. This, in turn, will promote the flow of financial resources to the agricultural sector and alleviate agricultural credit constraints. Therefore, this paper argues that there may be a non-linear relationship between county financial marketization reform and HQDAE. When the level of financial marketization in a county exceeds a certain threshold, the effect of financial marketization on the HQDAE changes from inhibitory to promotive.

County towns have a close relationship with agricultural economic development as they serve as a hub for contemporary agricultural production and industries, linking cities and villages [13]. In the past two years, the Chinese government has placed a strong emphasis on ’accelerating the construction of urbanization with counties as important carriers’. This is a strategic and forward-thinking approach to China’s new urbanization, and a crucial step towards breaking down the dual structure of urban and rural areas while promoting the HQDAE. County urbanization supports the industrial and economic base, population aggregation, and application scenarios necessary for the development of financial marketization [14]. In turn, financial marketization provides financing and development vitality for the construction of infrastructure and public goods during the process of urbanization [15]. These two processes are mutually reinforcing and complementary [16]. Therefore, what role does urbanization at the county level play in the interaction between financial marketization and HQDAE? Incorporating county urbanization into the analytical framework of financial marketization can provide a scientific decision base for promoting the high-quality development of China’s agricultural economy.

Academic literature on financial marketization, urbanization, and agricultural economic expansion can be broadly categorized into three groups. The first category focuses on the relationship between financial development and economic expansion. Relevant research in this area can be divided into two subcategories. On one hand, scholars have explored the economic implications of financial marketization from an urban economic perspective [17]. Encouraging capital deepening and technical advancement, financial marketization has a considerable positive impact on real economic growth. This impact also has regional spillover effects [18–20]. However, some scholars believe that excessive development of financial marketization may weaken its role in promoting economic growth, as seen during the East Asian Financial Crisis and the US Subprime Mortgage Crisis [21]. The relationship between financial marketization and the real industrial economy is complex and non-linear, rather than a simple linear one. The industrial economy benefits from a certain level of financial development, but excessive financial development or inhibition can put the real industrial sector at risk of the ’financial curse’ [22]. Meanwhile, scholars have studied the relationship between financial development and agricultural economic growth from the perspectives of financial scale, efficiency, structure, and innovation [23]. However, there is no consensus on the conclusion, which is mainly expressed as the positive promotion effect [24], negative inhibition effect [25], or a complex non-linear relationship [26].

The literature in the second category analyzes the impact of urbanization on agricultural economic growth, with two main perspectives. On one hand, some academics argue that urbanization has a positive effect on agricultural economic growth by increasing farmers’ incomes, fostering technological advancement in agriculture, increasing demand for agricultural products, and driving the restructuring and upgrading of the agricultural industry [27,28]. They also suggest that this effect becomes more significant over time [29]. However, the positive spillover effect also presents the problem of uneven regional development [30]. On the other hand, several researchers have concluded that urban development does not support agricultural economic growth and may even have a detrimental impact [31]. Urbanization reduces variables that contribute to agricultural productivity, such as agricultural labor and arable land area [32]. The impact of urbanization on agricultural economic growth varies geographically, with sub-developed regions experiencing a boost while developed and undeveloped regions see a dampening effect [33]. The literature’s third category focuses on the relationship between financial development and urbanization. Finance can provide capital for urbanization construction and improve the effectiveness of capital allocation for optimal allocation of relevant capital, achieving the goal of financial support for urbanization construction [34]. Financial marketization can also impact urbanization through knowledge spillover and industrial upgrading [35]. On the other hand, some academics have focused on the impact of urbanization on the development of the local financial industry. They argue that as infrastructure is built and commercial and industrial enterprises are developed, there will be a greater need for financial capital. This, in turn, will help the economy grow and the financial industry to develop [36].

This paper examines the relationship between county financial market development and HQDAE, focusing on the impact of different stages of development. It also explores the mechanism of county urbanization in this relationship. The paper then tests these theories empirically using county panel data and employing various models, including the panel fixed effect model, the panel threshold model, and the intermediary effect model. The study considers two dimensions of the agricultural economy: ’increasing quantity’ and ’improving quality’. County financial marketization has a non-linear impact on the HQDAE. The impact is ’U-shaped’ on the total growth of the agricultural economy and ’inverted N-shaped’ on the quality improvement of the agricultural economy. Furthermore, the relationship between county financial marketization and the HQDAE is influenced by the threshold effect of the level of county urbanization. As urbanization levels increase in a county, the impact of financial marketization on HQDAE also increases significantly. Furthermore, county financial marketization promotes urbanization and accelerates urban-rural integration, which in turn leads to HQDAE. This study highlights the significance of local governments at the county level in promoting a differentiated county financial system. During the early stages of financial market-oriented reform, the government should enhance financial services in rural areas by offering tax incentives, policy support, and other benefits to prevent financial resources from being redirected away from agriculture. In the later stages of financial marketization reform, the government should reinforce financial supervision to prevent the diversion of financial resources from industry to capital. To achieve HQDAE, it is necessary to promote financial market-oriented reform and accelerate urbanization in counties. This will help break the dual economic structure of urban and rural areas and promote the flow of financial capital, technology, and human capital from county cities to rural areas.

This paper aims to contribute to the research on the effects of county financial marketization on the agricultural economy. The majority of the literature on this subject focuses on the urban industrial economy at the provincial and municipal levels. Therefore, this paper fills a gap in the research by exploring the effects of county financial marketization on the agricultural economy. The limited literature on this topic also fails to consider any potential impact on the quality of the agricultural economy, focusing solely on its overall effects. This research builds a nonlinear analytical framework of financial marketization impacting the HQDAE, which improves the theoretical analysis in this area, using the unequal financial development at the county level as the entrance point.

Secondly, there is limited literature analyzing the internal logic between financial marketization and HQDAE from the perspective of county urbanization construction. Ignoring the role of the external variable of county urbanization, failing to effectively utilize the integration and development of county finance and urbanization to drive the sustainable agricultural economy, or both, could constrain the potential of HQDAE. This paper presents an analytical framework that combines financial marketization, county urbanization, and sustainable agricultural economy. It examines the influence of financial marketization on county urbanization, which in turn affects the HQDAE, from the perspective of the flow of urban-rural factors. The paper also analyzes the heterogeneity of financial marketization that impacts the high-quality growth of the agricultural sector under the threshold effect of county urbanization.

Most current quantitative studies on financial lending and the sustainable agricultural economy are based on data collected at the provincial and municipal levels or from cross-sectional surveys. This article, however, uses Meso-level county panel data to explore the relationship between financial marketization and the sustainable agricultural economy in terms of aggregate growth and quality improvement.

## 2 Theoretical analysis and research hypothesis

### 2.1 Mechanisms of the impact of county financial marketization on HQDAE

Financial liberalization is a key indicator of financial progress. Recently, the ’polarization effect’ of China’s county-level financial development has emerged, with certain developed counties reaching new peaks. However, some underdeveloped countries continue to struggle with high power consumption, low production capacity, difficult economic transformation, and limited financial resources. This has led to uneven financial development between regions [37,38]. It has been demonstrated that financial capital has the potential to both positively contribute to and inhibit economic growth. This paper makes the case that the impact of county financial marketization on high-quality development of agricultural economics also varies structurally, depending crucially on the degree of financial marketization.

When county financial marketization is low, there are fewer credit funds available to support the growth of the real economy. Agriculture is a high-risk, low-return sector with little collateral and greater information asymmetry, making it challenging for agricultural business entities to secure credit capital assistance for production [39]. Financial institutions are currently drawing rural funds through storage operations and moving them to more lucrative non-agricultural sectors or other counties with higher levels of economic development for their own gain. This has resulted in a serious lack of financial resources in the area of agricultural production. Additionally, widespread financial exclusion hinders the HQDAE by distorting financial markets and causing inefficient capital allocation.

When the county’s financial system reaches a certain level of development, it will encourage the high-quality development of the agricultural economy. First, the county’s financial market structure can be optimized through financial marketization development, which can also increase the reach and uptake of rural financial services. There will be a gathering of state-owned banks, joint-stock banks, city merchant banks, and other financial institutions in addition to the rural credit cooperatives, which predominate the county financial market. This gathering of different types of financial institutions aims to optimize the financial market’s structure and increase competition in the county financial market. According to the market power hypothesis, increased market competition will decrease the monopoly of traditional rural credit unions, allowing financial institutions to focus more on serving the ’long tail’ of customers, including small farmers and emerging agricultural entrepreneurs. The financial marketization in counties can have a ’radiation effect’ and ’spatial spillover effect’ that encourages the flow of financial resources from cities, towns, and non-agricultural industries to the agricultural sector. This can increase the penetration of rural finance and promote financial support for agriculture [40,41]. Additionally, the development of financial marketization can improve the effectiveness of the county’s financial capital allocation. The financial marketization of the county can have a ’network effect’ that reduces the cost of exchanging, searching, and sharing information. This can improve the effectiveness and manner of information dissemination between regional financial institutions and credit entities, thereby reducing the costs of financial transactions caused by uncertainty and information asymmetry during capital financing. By reducing the cost of pre-credit investigation and post-credit supervision, financial institutions can more effectively allocate capital, decrease the likelihood of credit mismatch, and provide more funding to needy agricultural businesses. Thirdly, in the context of the growth of the digital economy, such as the Internet and e-commerce, pooling financial resources in counties can lead to the emergence of new economies and businesses, stimulate credit demand from farmers, expand advanced agricultural production and service models, prompt the upgrading of production and consumption structures in rural areas, and ultimately provide more impetus for agricultural production and farmers to grow. Fourthly, financial marketization in the county can promote the spread and exchange of agricultural technology, as well as the diffusion of advanced production knowledge and management experience from non-agricultural sectors to rural areas, ultimately improving agricultural production efficiency and bringing about agricultural economic growth.

However, the positive contribution effect of the county’s financial marketization on the HQDAE will be weakened when it reaches a high level of development. This is due to the law of diminishing marginal utility of financial capital input factors [42]. The overabundance of financial institutions in the county will increase competition among them, leading to greater pressure for short-term profitability. As a result, some financial institutions may invest significant sums of money in the stock market and other capital markets to pursue regulatory arbitrage and speculative activities [43]. This can have a negative impact on real industries such as agriculture by reducing their capital input. Excessive competition among financial institutions can lead to financial resource mismatch, increasing the rate of non-performing bank loans and financial systemic risk [44]. However, when financial development reaches a high level, concentration of financial institutions and credit funds can promote the prosperity and development of non-agricultural industries. The non-farm sector’s dominant development will further marginalize agriculture through ’hollowing out’ and ’non-farming’ in rural areas, which will harm agriculture’s sustainable development.

In conclusion, the impact of county financial marketization on the HQDAE depends on the level of financial development. The expansion of the county’s sustainable agricultural economy will be aided by an increase in the level of financial marketization within a specific range, but it will be hindered by levels of financial marketization that are either too low or too high. Therefore, this paper proposes research hypothesis 1:

H1: The influence of county financial marketization on the HQDAE is nonlinear.

Due to differences in resource endowment, economic development, and the level of financial marketization in different regions, the impact of county financial marketization on the HQDAE is heterogeneous. Previous theoretical analysis suggests that the role of county financial marketization in different stages of development on the HQDAE varies. The level of county financial marketization can impact the HQDAE, with optimal levels promoting it and both low and high levels having a negative effect. Currently, the western region is in the early stages of financial marketization, with a small financial scale and significant financial exclusion in agricultural development. As a result, county financial marketization is dampening the HQDAE in the western region. The level of financial marketization in some counties in the central region is higher than that in the western region, but it has not reached the level of financial super-scale development. Therefore, county financial marketization has a non-linear effect on the HQDAE in the central region, following a ’U’ shape. In the eastern region, county financial marketization levels are generally higher than the national average. Some coastal counties have experienced financial super-scale development. However, financial overscale development can lead to a misallocation of resources and reduce financial investment in agriculture, which is not conducive to the HQDAE. Therefore, the county financial marketization in the eastern region has an inverted ’N’-shaped effect on the HQDAE. Therefore, we propose research hypothesis 2:

H2:County financial marketization has a varying impact on the HQDAE across different regions.

### 2.2 Analysis of threshold effect of county urbanization

The urban leadership theory posits that towns and cities serve as spatial carriers for industrial growth by providing the necessary infrastructure, application scenarios, and talent support [14,45]. Financial institutions face challenges in carrying out basic financial operations, such as researching, developing, and innovating agricultural credit products and constructing agricultural credit information databases during the early stages of county urbanization due to the lack of basic conditions, such as financial talent and bank branch establishment. The encouragement effect of county financial marketization on the high-quality growth of the agricultural sector is minimal. Furthermore, during this period, the main function of financial marketization development was to concentrate surplus rural funds to support the construction of county towns and industries [46]. This caused a shortage of rural financial supply, and financial marketization had a dampening effect on the growth of county agricultural economies.

The impact of financial marketization on the expansion of the county’s sustainable agricultural economy becomes evident once the level of urbanization in the county reaches a certain threshold. Urbanization’s diffusion effect has led to the proliferation and transfer of cutting-edge production elements, technologies, and organizational and management systems from urban regions to nearby rural areas [47]. The use of advanced production factors and technologies in agriculture is expected to lead to an increase in agricultural-related financial services. This, in turn, will prompt financial institutions to develop their agricultural-related services further. Urbanization is a complex and organized process that involves infrastructure building, investment in industrial and commercial enterprises, talent gathering, urban scale expansion, and innovation knowledge spillover. These factors are favorable to the gathering of financial institutions and the accumulation of financial capital [48], resulting in a continuous growth of financial resources that can be allocated to rural areas. County urbanization’s ’demand-led’ role will also promote the development of financial institutions’ business types and credit product innovation, thus improving the county’s financial system and enhancing the efficiency of financial support for agriculture. In summary, the development of county financial marketization affects the supply capacity and allocation effectiveness of agricultural-related funds. This impact varies depending on the level of urbanization in the county. Research hypothesis 3 was formulated based on this observation:

H3: The relationship between county financial marketization and the HQDAE is influenced by the threshold effect of county urbanization.

### 2.3 Analysis of intermediary effects of county urbanization

The analysis of the impact of financial marketization on urbanization in the county is divided into two main aspects. County finance can offer financial credit funds for urban infrastructure construction, which can optimize capital allocation and diversify risk. This can help businesses involved in constructing for-profit public goods during the urbanization process to ease financial constraints and investment risks, furthering county urbanization development. However, the agglomeration of production elements is the primary driving force behind the development of county towns. Financial capital plays a crucial role in triggering the agglomeration of production factors [49]. The financial services and capital provided by the financial market are necessary inputs for the functioning of businesses. These businesses are often located near financial institutions to reduce transportation and other costs, resulting in an initial impact on location and the creation of an industrial agglomeration. Industrial agglomeration and structural upgrading will generate new financial demand and encourage the aggregation of financial institutions in the county through the ’demand-led’ effect. This will result in a cyclical cumulative causal effect and support the ongoing growth of county towns [49].

The impact of county urbanization on HQDAE is significant. County urbanization draws laborers from rural areas to cities by improving public services and infrastructure, such as education, healthcare, culture, and recreation [50]. This, in turn, raises the income levels of farmers, which helps to alleviate the lack of capital for agricultural production and the lack of capital accumulation, ultimately leading to increased capital investment in agriculture. Secondly, county urbanization can improve the current situation of fragmented agricultural land management by encouraging rural labor transfer to non-agricultural employment [51]. This will allow for the integration and revitalization of the original scattered, idle, and inefficiently used land resources, promoting large-scale, industrialized, and organized land management. The urbanization of counties can lower the cost of agricultural production, boost labor and land productivity, and foster the HQDAE. Additionally, it helps to gather people with advanced knowledge and raise the level of regional human capital, promoting agricultural technology development and innovation by improving infrastructure conditions for technology development and manufacturing [52]. At the same time, urbanization in counties facilitates communication between urban and rural residents, leading to the introduction of urban management ideas, advanced production know-how, and experience into rural areas and the agricultural sector. This improves the overall standard of agricultural producers and encourages their adoption of cutting-edge technology and agricultural equipment through a radiation-driven effect, ultimately increasing agricultural output.

In conclusion, the financial marketization of counties promotes urbanization by providing funding for urban economic growth, population urbanization, and the construction of urban infrastructure and public services. County urbanization can also encourage the flow of factors between urban and rural areas, as well as knowledge spillovers, reallocating rural factor resources, and ultimately fostering HQDAE [33]. Based on this, we propose research hypothesis 4 in this work.

H4: County urbanization plays a mediating role in the relationship between county financial marketization affecting the HQDAE.

## 3 Methodology and data

### 3.1 Model setting

#### 3.1.1 Baseline regression model

This article uses the benchmark model shown below to examine the non-linear impact of county financial marketization on the HQDAE.


$$agri_{i,t}=\beta_{0}+\beta_{1}f\mathrm{in}a_{i,t}+\beta_{2}f\mathrm{in}a_{i,t}^{2}+\beta_{3}f\mathrm{in}a_{i,t}^{3}+\beta_{4}urb_{i,t}+\beta_{5}X_{i,t}+u_{i}+e_{t}+\varepsilon_{i,t}$$ (1)

In Eq (1), the level of HQDAE in the ith county (county-level city) in year t is represented by *agri*_*i*,*t*_. *fina*_*i*,*t*_ is the level of county financial marketization, $f\mathrm{in}a_{i,t}^{2}$ and $f\mathrm{in}a_{i,t}^{3}$ are the secondary and tertiary terms of county financial marketization respectively, *urb*_*i*,*t*_ is the level of county urbanization. In addition, *X*_*i*,*t*_ denotes a range of other control variables affecting HQDAE, *u*_*i*_ denotes area fixed effects, *e*_*t*_ denotes time fixed effects, *ε*_*i*,*t*_ is a random disturbance term, *β* is coefficients to be estimated. If *β*_1_ is significantly less than 0 and *β*_2_ is significantly greater than 0, then there is a positive "U-shaped" relationship between county financial marketization and HQDAE; If *β*_2_ is significantly greater than 0 and *β*_1_ and *β*_3_ are significantly less than 0, then there is an inverse ’N-shaped’ relationship between county financial marketization and HQDAE.

#### 3.1.2 Panel threshold model

Based on the analysis above, the impact of county financial marketization on HQDAE may be influenced by the threshold effect of county urbanization. The extent and direction of this impact may vary depending on the level of urbanization development. This study presents a panel threshold regression model based on Hansen’s threshold theory [53] to eliminate bias in the estimated results caused by arbitrary subjective partitioning of intervals. The model includes a threshold value and can be expressed as follows:

$$agri_{i,t}=\beta_{0}+\beta_{1}fina_{i,t}\cdot F(urb_{i,t}<\gamma_{1})+\beta_{2}fina_{i,t}\cdot F(urb_{i,t}\geq \gamma_{1})+\beta_{3}X_{i,t}+u_{i}+e_{t}+\varepsilon_{i,t}$$ (2)

This work expands the single-threshold model to create a multiple-threshold panel. model in light of the possibility of numerous thresholds for county urbanization:

$$\begin{array}{l} agri_{i,t}=\beta_{0}+\beta_{1}fina_{i,t}\cdot F(urb_{i,t}<\gamma_{1})+\beta_{2}fina_{i,t}\cdot F(\gamma_{1}\leq urb_{i,t}\\ \;<\gamma_{2})+\cdots \cdots +\beta_{n}fina_{i,t}\cdot F(\gamma_{n-1}\leq urb_{i,t}<\gamma_{n})+\beta_{n+1}fina_{i,t}\cdot F(urb_{i,t}\\ \;\geq \gamma_{n})+\beta_{n+2}X_{i,t}+u_{i}+e_{t}+\varepsilon_{i,t} \end{array}$$ (3)

In Eqs (2) and (3), the level of county urbanization (*urb*_*i*,*t*_) is the threshold variable, *γ* denotes the unknown threshold value and *F*(∙) is the indicator function, When *urb*_*i*,*t*_ satisfies the condition in the parentheses of the indicator function, then *F*(∙) = 1, otherwise *F*(∙) = 0. The other variables are explained in the same way as Eq (1). Whether there is a threshold effect of county urbanization on the impact of financial marketization on HQDAE, the original hypothesis is H0: *β*_1_ = *β*_2_, i.e. there is no threshold effect, and the alternative hypothesis is H1: *β*_1_≠*β*_2_. If the original theory is correct, Eq (2) becomes a linear equation since there is no threshold effect. The threshold effect is assumed to exist if the initial hypothesis is rejected, and thus opens the door to testing the second threshold, and so on.

#### 3.1.3 Mediating effects model

According to the research above, financial marketization can affect HQDAE through the variable county urbanization as a mediating factor. The study employs Edwards et al.’s technique to construct a mediating effect test model [54], which is better suited for evaluating the nonlinear relationship of variables than Wen Z. L. et al.’s ’Three-Step’ mediating effect test. The equations are as follows:

$$agri_{i,t}=\alpha_{0}+\alpha_{1}f\mathrm{in}a_{i,t}+\alpha_{2}f\mathrm{in}a_{i,t}^{2}+\alpha_{3}f\mathrm{in}a_{i,t}^{3}+\alpha_{4}X_{i,t}+u_{i}+e_{t}+\varepsilon_{i,t}$$ (4)

$$urb_{i,t}=\lambda_{0}+\lambda_{1}fina_{i,t}+\lambda_{2}fina_{i,t}^{2}+\lambda_{3}fina_{i,t}^{3}+\lambda_{4}X_{i,t}+u_{i}+e_{t}+\varepsilon_{i,t}$$ (5)

$$\begin{array}{l} agri_{i,t}=\eta_{0}+\eta_{1}fina_{i,t}+\eta_{2}fina_{i,t}^{2}+\eta_{3}fina_{i,t}^{3}+\eta_{4}urb_{i,t}+\eta_{5}fina\times urb_{i,t}\\ \;+\eta_{6}X_{i,t}+u_{i}+e_{t}+\varepsilon_{i,t} \end{array}$$ (6)

Specific test procedures: ①Test the impact of financial marketization and its multiple. terms. on HQDAE, focusing on the significance of coefficients *α*_1_, *α*_2_ and *α*_3_ in Eq (4). ②Focus on the sign of coefficients *λ*_1_ and *η*_4_ in Eqs (5) and (6) and their significance; if both coefficients *λ*_1_ and *η*_4_ are significant, this indicates a mediating effect; If at least one of *λ*_1_ and *η*_4_ is insignificant, then a further Sobel statistic needs to be constructed to test the significance of the product of the coefficients (i.e. whether the original hypothesis of *λ*_1_*η*_4_ = 0 is rejected), and if significant then this implies the presence of a mediating effect. The critical probability of the Sobel statistic can be determined by looking up the table of critical values proposed by [55].

### 3.2 Selection of variables

#### 3.2.1 Explained variables

HQDAE aims for qualitative improvement while maintaining reasonable quantitative growth. ’Economic Quantity’ represents the scope and degree of development, while ’Economic Quality’ reflects development efficiency and competitiveness. These two concepts are interconnected and have an impact on one another to support sustainable agricultural growth. Therefore, this paper selects indicators to measure the HQDAE from two perspectives: total growth and QIAE. Total agricultural economic growth (TGAE) is expressed as gross agricultural product in the county area. In general, total factor productivity (TFP) in agriculture is considered to be the driving force of economic quality improvement [56]. The purpose of this paper is to measure the quality improvement of the agricultural economy (QIAE) by using the total factor productivity (TFP) of agriculture. The DEA-Malmquist index method is employed to measure TFP, which avoids bias caused by improperly set production function forms and improves the accuracy of the findings. The Malmquist index formula for technical circumstances from period t to (t+1) is created as follows:

$$M_{i}(x^{t+1},y^{t+1},x^{t},y^{t})={\left[\frac{D_{i}^{t}(x^{t+1},y^{t+1})}{D_{i}^{t}(x^{t},y^{t})}\times \frac{D_{i}^{t+1}(x^{t+1},y^{t+1})}{D_{i}^{t+1}(x^{t},y^{t})}\right]}^{1/2}$$

Where, when *M*_*i*_>1, it represents agricultural productivity growth; *M*_*i*_ = 1, agricultural productivity is stable and constant; and *M*_*i*_<1 represents agricultural productivity decline. The Malmquist index measures the change in efficiency between two years by using input-output variables. In this study, 543 sample counties (county-level cities) with data from 2012 to 2018 were selected to measure the Malmquist total factor productivity index for agriculture from 2013 to 2018. The study utilized the value added of the primary sector (at constant 2012 prices) as the output indicator. The number of people employed in the primary sector was used as the labor input, while the area sown with crops was used as the land input. The total power of agricultural machinery was used as the mechanical power input, and the amount of fertilizer applied (converted) was used as the chemical fertilizer input. These input-output variables were selected based on existing literature [57].

#### 3.2.2 Core explanatory variables

The explanatory variable in this study is county financial marketization. The financial marketization index constructed by [58] is the most influential measure of financial marketization. However, this index only includes the financial marketization index by province from 1999 to 2009, which is not applicable to this study. The theory of financial deepening is at the core of financial marketization. It advocates for reducing excessive government intervention in finance and establishing the fundamental role of market mechanisms. Financial marketization is typically measured in terms of the size of financial assets relative to GNP, and its measurement can be summarized into two categories: monetization degree and financial-related ratio. The monetization share is mainly measured by M2/GDP, while the financial-related ratio uses the ratio of loan balances of financial institutions to GDP. The paper measures the level of financial marketization in counties by using the ratio of the year-end loan balance of financial institutions to GDP in each county area. This measurement is based on studies such as [8,59]. Meanwhile, this study introduces a new formula for the financial-related ratio metric that eliminates the influence of regional size disparities. The formula is as follows:

$$fina_{i,t}=\frac{loan_{i,t}/GDP_{i,t}}{\sum_{1}^{n}loan_{i,t}/\sum_{1}^{n}GDP_{i,t}}$$

Of which, *fina*_*i*,*t*_, *loan*_*i*,*t*_ and *GDP*_*i*,*t*_ represent the level of county financial marketization, the size of bank loans and the gross product of the i-th county region at period t. $\sum_{1}^{n}loan_{i,t}$ and $\sum_{1}^{n}GDP_{i,t}$ represent the total value of bank loans and the gross product of the n-th county region at time t, respectively.

#### 3.2.3 Mediating and threshold variables

The level of urbanization in the county is used as both a mediating and threshold variable in this study. The ratio of the resident urban population to the overall regional population is employed to depict the level of urbanization since the data on the urbanization rate is precise, exhaustive, and authoritative.

#### 3.2.4 Control variables

Considering the influence of other factors on the HQDAE, the following variables are used as control varia-bles in this paper based on relevant studies. ①Industrial structure: The level of resource support for economic sectors and the quality of economic development are both influenced by the industrial structure [60]. The growth of the non-agricultural sector can enhance agricultural capital. However, sustained agricultural development is impossible if industrial-agricultural and urban-rural linkages are not effectively managed. In this study, we use the contribution of secondary industry value added to regional GDP as a proxy for changes in industrial structure in county regions. ②Human capital is a key element that influences economic growth. This research follows the methodology used by Feng L. et al. [61] and employs the proportion of junior secondary school enrollment to the district’s overall population to describe the level of human capital at the county level. ③The level of agricultural scale operation is measured by the ratio of crop sown area to the number of employees in the primary industry. ④Resident consumption: Consumer goods total retail sales are used to calculate the amount of resident consumption. ⑤Government participation in economic activities: In China, local administrations are crucial to the growth of the sustainable agricultural economy. The ratio of local government fiscal spending to local GDP is used to illustrate local government engagement in economic activity, drawing on Huang H. G. et al. [7]. ⑥Population density: Population density is measured by calculating the ratio of the total population of the county area to the area of the administrative district.

### 3.3 Data sources

#### 3.3.1 Data sources and descriptions

In this paper, the data of 543 counties (county-level cities and banners) in China from 2013–2018 were finally selected as the research sample, which are balanced panel data. The considerations mainly include: firstly, some county areas have not published county urbanization rate indicators and related rural economic development indicators, such as total agricultural machinery power and discounted agricultural fertilizer. Secondly, the disclosure period is relatively short, and the indicator data are seriously missing. It is important to note that China’s municipal districts exhibit distinct urban economic characteristics and differ significantly from counties (including county-level cities) in terms of their economic and financial features, as well as the allocation of financial power and responsibilities. Therefore, they are not included in the statistics. Due to ongoing economic development, county-level administrative divisions in China have undergone frequent adjustments and changes in recent years. Therefore, we have excluded a small number of counties that were divided into districts due to the removal of counties in the selection of the county sample. Additionally, we have excluded Beijing, Shanghai, Tianjin, Tibet Autonomous Region, and the Hong Kong, Macao, and Taiwan regions of China.

The sample data’s representativeness is described as follows: Firstly, the sample areas were mostly chosen from functional categories since China’s agricultural economic development is primarily concentrated on the main grain-producing regions and the balanced production and marketing regions. Secondly, there is considerable variation in the degree of urbanization and financial development in the sample locations. These traits are present throughout the entire nation. Additionally, the sample areas were selected from various geographical regions due to the clear variation in the level of regional economic growth in different geographic locations.

It is important to note that the sample period for the study was chosen to be between 2013 and 2018. At the beginning of the twenty-first century, China’s county financial markets were characterized by ’financial inhibition,’ and the predominant financial institutions were rural credit unions. Since 2006, the rural financial market in Chinese counties has been gradually liberalized. As a result, various new rural financial institutions, such as village banks, lending firms, and mutual societies, have entered the market. However, the selection of a site and the design of a financial institution’s store can be time-consuming. During the two years leading up to and following the 2008 financial credit crisis, the financial institutions’ credit risk increased. This hindered the expansion of financial credit in China’s counties and, to some extent, the plans of all types of financial institutions to set up new business outlets. After a period of development and risk resolution, the number of financial institutions, product types, credit scales, and market mechanisms in China’s counties has increased, and the level of financial marketization has continued to rise. The empirical data in this paper is based on the urbanization rate indicators from 2013. The China Bureau of Statistics altered the method of calculating gross product in 2018, resulting in some discrepancies in the value of gross agricultural product in each county-level city district (county) in China before and after 2019. Therefore, the research sample in this paper is based on data from 2018 to maintain consistency and coherence.

The empirical data were primarily sourced from the China Statistical Yearbook, China County Statistical Yearbook, Wind database, EPS database, the statistical database of China Economic Network, and the statistical bulletin of each county (county-level city) in the relevant provinces. To handle missing or outlier data, the following processing instructions were applied: We excluded the sample of counties with more than two consecutive years of missing data. Additionally, we supplemented individual missing data with information from statistical yearbooks of the provinces, cities, and districts where the sample counties, county-level cities, and banners are located. Third, we utilized linear interpolation and extrapolation to smooth the data of individual indicators that are still missing. Finally, this study employs the non-censored bilateral 2% tail reduction approach to address the outliers of specific variables and reduce the impact of sample outliers on the estimated results. Additionally, the relevant nominal economic indicators are deflated using the provincial GDP price deflator in the sample counties to accurately portray economic growth.

#### 3.3.2 Descriptive statistics

Table 1 displays the descriptive statistics for the variables of interest.

**Table 1:** Descriptive statistics of variables.

Variable type	Variable symbols	Definition of variables	Sample size	Average	Standard deviation
Explained variables	agdp	TGAE	3258	2.850	0.977
	tfp	QIAE	3258	1.261	0.533
Core explanatory variables	fina	Level of county financial marketization	3258	1.031	0.565
Intermediary/threshold variables	urb	County urbanization rate	3258	0.415	0.099
Control variables	scale	Level of agricultural scale operation	3258	5.723	3.075
	sale	Total retail sales of social consumer goods	3258	3.745	1.091
	indust	County industry structure	3258	0.459	0.143
	hum	Level of human capital	3258	0.049	0.016
	popu	Population density	3258	0.039	0.027
	gov	Government economic participation	3258	0.298	0.328

To ensure the validity of the estimated parameters of the model, we analyzed the panel data for stationarity using the LLC test, IPS test, Fisher-ADF test, and Fisher-PP test. The results of the tests are presented in Table 2, indicating that all variables are smooth series.

**Table 2:** Stationarity test results of variable data.

Test Methods	LLC	HT	Fisher-ADF	Fisher-PP	conclusions
agdp	-17.745*** (0.000)	-18.681*** (0.000)	408.351*** (0.000)	432.523*** (0.000)	smoothly
tfp	-31.047*** (0.000)	-32.487*** (0.000)	432.523*** (0.000)	432.523*** (0.000)	smoothly
fina	-28.614*** (0.000)	-30.111*** (0.000)	432.523*** (0.000)	432.523*** (0.000)	smoothly
urb	-23.640*** (0.000)	-24.906*** (0.000)	432.523*** (0.000)	1432.523*** (0.000)	smoothly
hum	-21.863*** (0.000)	-23.160** (0.000)	432.523*** (0.000)	432.523*** (0.000)	smoothly
scale	-18.880*** (0.000)	-20.111*** (0.000)	411.028*** (0.000)	432.523*** (0.000)	smoothly
indust	-29.213*** (0.000)	-31.179*** (0.000)	432.523*** (0.037)	432.523*** (0.000)	smoothly
sale	-21.173*** (0.000)	-21.793*** (0.000)	432.523*** (0.000)	432.523*** (0.000)	smoothly

Note: The HT test reports results as Z-values, the rest of the tests report results as T-statistic values, and P-values for statistical tests are in parentheses. ***, ** and * indicate significant at the 1%, 5% and 10% levels respectively.

## 4 Analysis of empirical results

### 4.1 Analysis of benchmark estimation results

This work utilizes OLS mixed regression, random effects models, and two-way fixed effects models to conduct econometric tests. The estimated results are presented in Table 3. Columns (1) and (5) show the estimates of Eq (1) using OLS mixed regression without any additional control variables. When examining the growth of agricultural economic scale, it was found that financial marketization and its quadratic term have a significant impact at the 1% level. The coefficient for the primary term is negative, while the coefficient for the secondary term is positive. This suggests that financial marketization has a significant positive impact on the TGAE in the county, following a non-linear ’inhibiting, then promulgating’ relationship. The coefficients for primary, secondary, and tertiary financial marketization all pass the 5% significance level test when agricultural economic quality is the explanatory variable. The primary and tertiary terms are negative, while the secondary terms are positive. This suggests that county financial marketization has a significant inverse ’N-shaped’ effect on the improvement of agricultural economic quality. This study employed an OLS mixed regression model to investigate the impact of county urbanization development on the growth of agricultural economies in terms of size (refer to column 2) and quality (refer to column 6). The results indicate that county urbanization development benefits the HQDAE, which is consistent with the findings of other researchers [29]. Next, we introduce control variables and examine the impact of regional and temporal differences on the regression estimation process. We then test these differences again using random effects models (refer to columns (3) and (7)) and two-way fixed effects models (refer to columns (4) and (8)). The results of the estimation indicate that the coefficients of the primary, secondary, and tertiary terms of county financial marketization are consistent with columns (1) and (5), which confirms the reliability of the findings. Furthermore, the following estimates will mostly be based on a two-way fixed effects model as the Hausman test suggests that two-way fixed effects are better estimated than random effects.

**Table 3:** Results of the baseline model estimation.

Variables	Explained variable: TGAE				Explanatory variable: QIAE			
	OLS OLS		RE	FE	OLS OLS		RE	FE
	(1)	(2)	(3)	(4)	(5)	(6)	(7)	(8)
fina	-0.089*** (0.015)		-0.125*** (0.022)	-0.099*** (0.023)	-0.071** (0.028)		-0.143*** (0.036)	-0.071* (0.040)
fina^2	0.007*** (0.002)		0.011*** (0.002)	0.009*** (0.002)	0.036*** (0.013)		0.053*** (0.017)	0.038** (0.018)
fina^3					-0.003** (0.001)		-0.003** (0.001)	-0.003* (0.002)
urb		1.850*** (0.191)	-1.717*** (0.200)	-0.151 (0.367)		0.622* (0.358)	1.981*** (0.257)	1.217** (0.596)
hum			0.141 (0.558)	0.066 (0.583)			-1.307 (0.879)	-2.914*** (0.998)
scale			0.020*** (0.004)	0.023*** (0.004)			0.000 (0.005)	0.005 (0.007)
indust			-0.247*** (0.074)	-0.243*** (0.081)			-0.789*** (0.116)	-0.416** (0.163)
sale			0.513*** (0.026)	0.134*** (0.041)			0.032 (0.024)	-0.164** (0.064)
constant	0.875*** (0.079)	2.082*** (0.080)	-1.795*** (0.123)	-0.578*** (0.143)	0.811*** (0.093)	0.386 (0.251)	1.698*** (0.127)	2.053*** (0.246)
The individual effect	YES	NO	NO	YES	YES	YES	NO	YES
Time effect	YES	NO	NO	YES	YES	YES	NO	YES
F-test	2740.93	93.70		90.67	122.32	117.22		39.71
Wald Chi2			918.16				332.54	
R-squared	0.992	0.035	0.732	0.557	0.899	0.899	0.238	0.293
Sample size	3258	3258	3258	3258	3258	3258	3258	3258

Note: ***, ** and * indicate significant at the 1%, 5% and 10% levels respectively. Robust standard errors for clustering are in parentheses. Same below.

The empirical findings examine the study hypothesis H1 and demonstrate that county financial depth has a significant non-linear impact on HQDAE. The coefficients of the control variables align with theoretical expectations and are consistent with the findings of other scholars [57]. During the examined sample period, it was found that scale operation and residential consumption significantly contributed to county HQDAE. However, upgrading the county’s industrial structure had a significant negative impact on county HQDAE. The development of the secondary and tertiary sectors was found to take too many resources away from agricultural production, which negatively affected the development of the agricultural sectors. Furthermore, it should be noted that human capital also has a negative impact on HQDAE. This may be due to the fact that individuals with higher levels of education in rural areas are more likely to pursue opportunities outside of traditional agricultural work, such as starting non-farm businesses or seeking employment elsewhere.

### 4.2 Endogeneity discussion and robustness test

Several discussions on endogeneity and robustness tests were conducted in this study to reinforce the conclusions. The results are presented in Table 4. Firstly, the panel instrumental variable model (IV-2SLS) was used to address the potential endogeneity issue arising from the mutual causality between HQDAE and county financial marketization, which could lead to biased and inconsistent parameter estimation results. This study employs a one-period lagged county financial marketization as the instrumental variable, calculated using two-stage least squares (refer to columns (1) and (4)). The table shows that all under-recognition test p-values are zero and weak instrumental variable test F-values exceed the general standard (F-value of 10). The hypothesis of weak instrumental variables is rejected, indicating that the selection of instrumental variables is valid overall. The coefficients of county financial marketization and its quadratic term are consistent with the results of the benchmark regression and both pass the 1% significance test, indicating that the benchmark regression is robust after the endogeneity test. Secondly, control variables were added. To reduce the impact of omitted variables on the estimation results, this paper includes and re-estimates other variables that may affect the HQDAE, such as government economic activity participation and population density (refer to columns (2) and (5)). The coefficients for county financial marketization and its quadratic term are consistent with the results of the benchmark regression and both pass the 5% significance test. This suggests that the results of the benchmark regression are robust after the addition of control variables. Finally, the measurement of core explanatory variables has been replaced. Based on [38] research, this paper introduces a new core explanatory variable by regressing the ratio of loan balances of financial institutions to local GDP in county areas (see columns (3) and (6)) to measure the level of financial marketization. The coefficients for county financial marketization and its quadratic term are consistent with the benchmark regression results and both pass the 1% significance test. This suggests that the benchmark regression results are robust even after conducting endogeneity and robustness tests with replacement of explanatory variables. In conclusion, the endogeneity and robustness tests confirm the robustness of the benchmark regression results.

**Table 4:** Estimation results of robustness tests.

Variables	Explained variable: TGAE			Explanatory variable: QIAE		
	(1)	(2)	(3)	(4)	(5)	(6)
fina	-0.217*** (0.022)	-0.098*** (0.023)	-0.133*** (0.037)	-0.521*** (0.136)	-0.079** (0.039)	-0.095 (0.064)
fina^2	0.016*** (0.003)	0.009*** (0.002)	0.018*** (0.005)	0.233*** (0.063)	0.042** (0.018)	0.090* (0.045)
fina^3				-0.017*** (0.005)	-0.003** (0.001)	-0.011* (0.005)
urb	-2.736*** (0.168)	-0.122 (0.367)	-0.143 (0.359)	-0.218 (0.225)	1.328** (0.601)	1.335** (0.601)
hum	-0.155 (0.543)	-0.114*** (0.576)	-0.239 (0.571)	2.231*** (0.807)	-3.221*** (1.009)	-3.278*** (1.012)
scale	0.005** (0.002)	0.022 (0.004)	0.021*** (0.004)	0.014*** (0.003)	0.004 (0.006)	0.005 (0.006)
indust	-1.769*** (0.082)	-0.253*** (0.081)	-0.235*** (0.079)	-0.529*** (0.104)	-0.419** (0.162)	-0.406** (0.161)
sale	0.911*** (0.011)	0.132*** (0.039)	0.124*** (0.039)	-0.007 (0.016)	-0.166** (0.064)	-0.171*** (0.064)
gov		0.012 (0.027)	0.006 (0.026)		0.121** (0.053)	0.114** (0.053)
popu		-2.841** (1.228)	-0.086** (0.036)		-2.203** (1.005)	-0.068** (0.030)
constant	-2.349*** (0.078)	-0.438*** (0.145)	2.314*** (0.135)	1.725*** (0.216)	2.135*** (0.257)	2.056*** (0.246)
Individual effect	YES	YES	YES	YES	YES	YES
Time effect	YES	YES	YES	YES	YES	YES
F-test	957.36	77.29	77.24	16.01	34.71	31.23
P-value for the underidentification test	0.000			0.000		
F-values for weak instrumental variable tests	57.669			82.749		
R-squared	0.773	0.388	0.359	0.841	0.295	0.295
Sample size	2715	3258	3258	2715	3258	3258

### 4.3 Regional heterogeneity analysis

The rate and level of agricultural economic growth vary significantly among counties due to differences in resource endowment, financial maturity, degree of urbanization, and other factors unique to each county. This paper divides the 543 county regions into three regions—eastern, central, and western—based on their province location and estimates them using a two-way fixed effects model based on panel data from each region. Table 5 presents the results, which reveal the heterogeneity of the impact of financial marketization on county agricultural economic growth across different regions.

**Table 5:** Regression results for different regions.

Variables	Explained variable: TGAE			Explanatory variable: QIAE		
	Eastern Region	Central Region	Western Region	Eastern Region	Central Region	Western Region
	(1)	(2)	(3)	(4)	(5)	(6)
fina	-0.236*** (0.064)	-0.143*** (0.034)	-0.007*** (0.002)	-0.180** (0.077)	-0.144* (0.075)	0.010 (0.012)
fina^2	0.171*** (0.048)	0.026** (0.013)		0.188*** (0.054)	0.073*** (0.026)	
fina^3	-0.036*** (0.009)			-0.0366*** (0.011)		
urb	0.279 (0.578)	-0.646 (0.718)	0.278 (0.260)	1.650* (0.906)	-1.463 (1.979)	1.047** (0.531)
Control variables	YES	YES	YES	YES	YES	YES
constant	-0.441*** (0.254)	-0.803*** (0.211)	-0.232** (0.108)	1.078*** (0.339)	2.871*** (0.586)	0.911*** (0.218)
The individual effect	YES	YES	YES	YES	YES	YES
Time effect	YES	YES	YES	YES	YES	YES
F-test	31.89	39.02	274.63	16.62	18.60	31.49
R-squared	0.334	0.784	0.816	0.349	0.377	0.298
Sample size	1062	1206	990	1062	1206	990

The regression results for the TGAE indicate that financial marketization has a complex non-linear impact on the sustainable agricultural economy in various regions. However, the nature of this non-linear effect varies, with a significant inverse ’N’-shaped relationship in the eastern region (refer to column (1)). Eastern China has a higher degree of financial development than the rest of the country. Based on the analysis presented in the previous article, the misallocation of resources resulting from the larger scale financial marketization has led to a decline in financial investment in the agricultural sector. This has significantly reduced its ability to support the sustainable growth of the agricultural economy on a larger scale, and may even have a suppressive effect. The correlation between the two variables shows a significant positive ’U’-shaped association in the central region of China, which is consistent with the overall regression results (refer to column 2). The financial marketization of counties in China’s western region has a significant negative impact on the TGAE (refer to column 3). The effect of county financial marketization on the improvement of agricultural economic quality also varies by region, as shown by the regression findings. The relationship between the two is inverted ’N’ type in the eastern region, which is consistent with the overall regression results. In the central region, there is a positive ’U’-shaped relationship. However, this relationship is not significant in the western region, indicating that the optimal allocation of financial resources in the Western region is inefficient.

### 4.4 Analysis of the threshold effect of county urbanization

This paper employs a panel threshold effect model to investigate whether the relationship between county financial marketization and HQDAE is affected by the threshold effect of county urbanization. The asymptotic distribution of the F-statistic was simulated using Bootstrap with 300 iterations of the triple threshold setting. The results are presented in Table 6. When the explanatory variable is the TGAE, the F-statistic P-value for the single threshold test is 0.0467, which is below the 5% significance level. The double threshold test did not pass the significance test, indicating that the impact of county financial marketization on the TGAE is affected by the single threshold effect of urbanization. Similarly, when the explanatory variable is the QIAE, the effect of county financial marketization on the improvement of the QIAE is influenced by the double threshold effect of urbanization. The threshold estimates are 0.3890 and 0.4926, respectively.

**Table 6:** Results of the threshold effect test for county urbanization.

**Explained variable: TGAE**				
**Type of threshold**	**F-statistic**	**P-value**	**Threshold**	**95% confidence interval**
Single-threshold test	51.06	0.046	0.362 ——	(0.361, 0.362)
Double threshold test	20.39	0.360		——
**Explanatory variable: QIAE**				
**Threshold type**	**F-statistic**	**P-value**	**Threshold**	**95% confidence interval**
Single threshold test	115.82	0.003	0.389	(0.387, 0.389)
Double threshold test	54.42	0.063	0.492	(0.489, 0.493)
Triple threshold test	37.25	0.880	——	——

Table 7 shows the estimates of the threshold effect based on the above tests. Column (1) indicates that when the urbanization level is below the threshold of 0.3620, the effect of county financial marketization on the TGAE is significantly negative with a coefficient of -0.0598. When the urbanization level is above the threshold of 0.3620, the negative effect becomes -0.0218. In summary, the relationship between county financial marketization and the QIAE is affected by the level of urbanization. As the level of urbanization increases, the inhibiting effect of financial marketization becomes less significant.

**Table 7:** Regression estimation results of the threshold effect of county urbanization.

Variables	Explained variable: TGAE		Explanatory variable: QIAE	
	(1)		(2)	
fina	urb≤0.362	-0.059***(0.007)	urb≤0.389	-0.065***(0.014)
	urb>0.362	-0.022***(0.007)	0.3890<urb≤0.492	0.037**(0.015)
	——	——	Urb>0.492	0.132***(0.019)
Control variables	YES		YES	
F-test	95.10		65.75	
R-squared	0.174		0.145	
Sample size	3258		3258	

From column (2), there are three intervals of variation in the coefficient of influence of county financial marketization on the improvement of the TGAE, with an overall positive ’U-shape’. When the level of urbanization is below the threshold of 0.3890, county financial marketization has a significant inhibitory effect on the QIAE, with a coefficient of -0.0655. When the level of urbanization is between the threshold of 0.3890 and 0.4926, county financial marketization plays a positive contributing role with a coefficient of 0.0378. The coefficient rises to 0.1320 when the level of urbanization is above the threshold of 0.4926, indicating the strongest promotion effect. County finance allocates more funds to urban construction when the level of urbanization is below a certain threshold (0.3890), resulting in underinvestment in the agricultural sector. Meanwhile, the county’s efforts to support the high-quality growth of the agricultural sector through financial marketization have a negligible or even suppressive impact due to imperfect capacity to disburse money and the layout of financial networks resulting from the lack of fundamental prerequisites. As the level of urbanization in the county increases, the infrastructure and industrial layout will gradually improve. Financial institutions not only gather and accumulate financial capital, but also promote the expansion and transfer of advanced production factors, technology, and innovative knowledge from urban areas to surrounding rural areas through the network effect and diffusion effect of urbanization. As a result, the resources that can be allocated by finance continue to increase, and the role of county financial marketization for HQDAE becomes more and more apparent. The findings support a non-linear relationship between county financial marketization and the HQDAE. Additionally, the relationship is influenced by the county urbanization threshold effect, which tests research hypothesis H2.

## 5 Analysis of mechanism testing

### 5.1 Analysis of the mediating mechanisms of county urbanization in the relationship between financial marketization affecting the HQDAE

Table 8 presents the results of the test for mediating effects of county urbanization. The empirical analysis conducted above, along with columns (1) and (4) in Table 8, confirms the significance of the coefficients of the primary, secondary, and tertiary terms of county financial marketization. Therefore, this section focuses on the signs of coefficients λ_(1) and η_4 in Eqs (5) and (6) and their significance. The regression results of total agricultural economic growth show that both the primary and secondary terms of county financial marketization in column (2) are significant at the 1% level. However, county urbanization in column (3) is not significant. To determine the significance of the mediating effect of county urbanization, the Sobel test is necessary. The Sobel test z-statistics for the primary and secondary terms of financial marketization are 1.47 and 1.48, respectively. Both values exceed the critical value of 0.97 at the 5% significance level, indicating a significant mediating effect of urbanization at the 5% level. The above information demonstrates that county financial marketization has a nonlinear effect on urbanization development and may impact the expansion of the agricultural sector through the intermediate function of urbanization. The regression results for agricultural economic quality show that the primary, secondary, and tertiary terms of county financial marketization in column (5) pass the 1% significance test. Additionally, the coefficient of urbanization in column (6) is also significant, tentatively indicating the existence of the mediating effect of urbanization. Furthermore, the results of the Sobel test indicate that urbanization serves as an intermediary channel for county financial marketization to influence the QIAE. Thus, it can be concluded that the convergence effect between county financial marketization and urbanization significantly contributes to the HQDAE, supporting research hypothesis H4.

**Table 8:** Estimated results of mediating effects.

Variables	Explained variable: TGAE				Explanatory variable: QIAE		
	agdp	urb		agdp	tfp	urb	tfp
	(1)	(2)		(3)	(4)	(5)	(6)
fina	-0.100*** (0.0101)	0.006*** (0.001)		-0.099*** (0.011)	-0.061** (0.025)	0.008*** (0.001)	-0.061** (0.025)
fina^2	0.009*** (0.001)	-0.001*** (0.000)		0.008*** (0.002)	0.035*** (0.013)	-0.003*** (0.001)	0.022 0(.014)
fina^3					-0.003** (0.001)	0.000*** (0.000)	-0.001 (0.001)
urb				-0.186 (0.120)			1.203*** (0.330)
fina*urb				-0.095* (0.054)			0.3486*** (0.123)
Control variables	YES	YES		YES	YES	YES	YES
Individual effects	YES	YES		YES	YES	YES	YES
Time effect	YES	YES		NO	YES	YES	YES
F-test	109.34	1939.97		124.44	91.78	1785.14	80.78
R-squared	0.307	0.887		0.269	0.289	0.888	0.295
Sample size	3258	3258		3258	3258	3258	3258
Sobel test for Z-statistic	one item 1.47**		Secondary term 1.38**		One item 3.07***	Secondary term 2.77***	Triple term 1.75***
Hypothesis validated or not	Yes				Yes		

Note: According to the table of critical values provided by MacKinnon (2002) (available from http://www.doc88.com/p-0973999637797.html), a z-statistic of greater than 0.97 for the Sobel test means significant at the 5% level and greater than 1. 656 means significant at the 1% level.

### 5.2 The heterogeneous impact of county urbanization on the HQDAE under the threshold of financial marketization

The intermediate effect of county urbanization may exhibit various stages in various zones depending on the level of financial marketization, the degree of technological development, and the industrial structure. This paper employs a panel threshold regression model to investigate whether the impact of county urbanization on the HQDAE is affected by the threshold effect of county financial marketization. The results of the threshold effect test are presented in Table 9. The impact of county urbanization on the TGAE is influenced by the double threshold effect of county financial marketization, with threshold estimates of 0.6057 and 1.2516, respectively, at a 95% confidence interval. The role of the QIAE is affected by the single threshold effect of county financial marketization, with a threshold estimate of 1.0948 at a 95% confidence interval. Table 10 displays the estimates of the threshold effects based on the above tests. The regression findings on the economic growth of agriculture demonstrate that as the financial depth of a county increases, the positive impact of urbanization on the total agricultural economic growth eventually decreases. Based on the regression analysis presented in Table 10, it can be observed that once the level of financial marketization surpasses the threshold of 1.0948, county urbanization has a positive impact on the improvement of agricultural economic quality. However, it is important to note that the coefficient decreases from 3.0840 to 2.9410, indicating a marginal decrease.

**Table 9:** Results of the threshold effect test.

**Explained variable: TGAE**				
**Threshold type**	**F-statistic**	**P-value**	**Threshold**	**95% confidence interval**
Single threshold test	54.81	0.000	0.605	(0.599, 0.607)
Double threshold test	45.96	0.000	1.2516	(1.239, 1.253)
Triple threshold test	14.44	0.463	——	——
**Explanatory variable: QIAE**				
**Threshold type**	**F-statistic**	**P-value**	**Threshold**	**95% confidence interval**
Single threshold test	20.55	0.066	1.095	(1.076, 1.098)
Double threshold test	9.56	0.536	——	——

**Table 10:** Results of estimating panel threshold effects.

Variables	Explained variable: TGAE		Explanatory variable: QIAE	
	(1)		(2)	
urb	fina≤0.605	1.096***(0.116)	fina≤1.095	3.082***(0.231)
	0.606< fina≤1.251	0.954***(0.117)	fina>1.095	2.941**(0.227)
	fina >1.251	0.827***(0.125)	——	——
Control variables	YES		YES	
F-test	43.61		43.19	
R-squared	0.232		0.250	
Sample size	3258		3258	

## 6 Conclusions and discussion

This paper presents an empirical analysis of the impact of county financial marketization on the HQDAE and regional differences using panel data from 543 counties (county-level cities) in China between 2013 and 2018. Additionally, we analyze the transmission mechanism of ’financial marketization—urbanization—the HQDAE’ using a mediating effects model. The study’s results are as follows:

The impact of county financial marketization on HQDAE has complex non-linear characteristics, with a ’U-shaped’ effect on TGAE and an ’Inverted N-shaped’ effect on QIAE. QIAE is negatively affected when the level of county financial marketization is below 1.4135 or greater than 7.0309. However, when the level of county financial marketization is between 1.4135 and 7.0309, it promotes QIAE. After conducting robustness testing, which addresses endogeneity problems using IV-2SLS, adds control variables, and replaces key explanatory variable measurements, the analysis still shows a non-linear relationship. This finding is consistent with previous research that suggests the link between financial capital and the actual industrial sector is complex and non-linear, rather than a simple linear relationship [10,21,22]. During the initial phases of financial marketization, county financial institutions invested funds in the industrial and commercial sectors due to their higher profit margins. This resulted in a significant loss of financial resources in the agricultural sector, leading to strong credit constraints on agricultural production. Such constraints are not conducive to the HQDAE. When the level of financial marketization in a county reaches a certain threshold, it intensifies competition in the county’s financial market. This prompts banks to expand the scope of their financial services to rural areas and increase the rural credit market to reach a wider customer base. This helps to meet the demand for financial services from farmers. This promotes agricultural capital investment and improves the consumption capacity of the rural market and agricultural production capacity, which has a positive effect on the HQDAE. However, when county finance develops on an oversized scale, unhealthy and disorderly competition among financial institutions can crowd out the financial supply of the agricultural sector, which is not conducive to HQDAE. Therefore, it is important to have an objective understanding of the impact of county financial marketization on the HQDAE. Moderate development of county financial marketization can aid the HQDAE, but it is crucial to remember that excessive or insufficient development can hinder it.

After examining the heterogeneity by region, it was found that county financial marketization has a significant effect on the HQDAE in the eastern region, following an inverse N-shaped pattern, while in the central region, it has a significant U-shaped effect. In the western region, county financial marketization has a significant inhibitory effect on the TGAE, but no significant effect on QIAE. Possible reasons for this include the fact that county financial marketization in western China is still in its early stages and lags behind the national average. Financial scale is limited, and agricultural development faces significant financial exclusion, which hinders the TGAE.

The test for the threshold effect of county urbanization reveals that the impact of county financial marketization on the HQDAE increases steadily. This effect is particularly pronounced on the QIAE as county urbanization levels rise. As urbanization increases in the county, it will lead to the expansion and transfer of advanced production factors, technology, and innovative knowledge from urban areas to the surrounding rural areas. This will result in a continuous increase in the resources that can be allocated by finance due to the network and diffusion effects.

The test on the mediation mechanism reveals that county urbanization significantly mediates the relationship between county financial marketization and the growth of a high-quality agricultural sector. This finding highlights the significance of aligning county finance with urbanization and promoting their integration to achieve a synergistic effect when advocating for the deepening reform of county finance and encouraging the return of county rural financial institutions to their roots in supporting the development of the local agricultural sector. Additionally, the urbanization driven by county financial marketization exhibits a non-linear characteristic with a diminishing marginal effect on promoting HQDAE. Financial resources may have an ’incentive’ effect during the early stages of county financial marketization, encouraging urbanization. This, in turn, can facilitate the diffusion of advanced technology, knowledge, and financial capital to rural areas, leading to improved technical efficiency in agricultural production [62]. This has shown a significant positive effect in promoting agricultural economic growth. When the financial overscale of a county grows, financial resources may have a ’crowding out’ impact. County financial institutions may prioritize speculating or engaging in regulatory arbitrage over industrial restructuring and urban building and development, which weakens the role of county urbanization in promoting high-quality agricultural economic development [43].

Based on these conclusions, we propose the following policy implications: county-level local governments should promote a differentiated county financial system. In the early stages of financial market-oriented reform, the government should formulate relevant incentive policies and reward methods, such as tax rebates, interest subsidies, and financial fund deposits for county financial institutions that support agricultural development. This will guide financial support for agricultural development. In the late stage of financial marketization reform, particularly in areas with high levels of financial development, the government should strengthen financial supervision to prevent the transfer of financial resources from the real industry to the capital market for the sake of chasing short-term profits. This can lead to a mismatch of financial resources, which is not conducive to agricultural development. To achieve the HQDAE, it is essential to promote county financial market-oriented reform and integrate finance with various fields in county cities. This will enable the optimal allocation of financial, technical, and human resources to rural areas through the platform supporting role of county towns, comprehensively promoting the HQDAE. Finally, county financial institutions should establish the innovative concept of serving the agricultural industry and comprehensively support rural revitalization and agricultural modernization. This can be achieved by strengthening the innovation of rural financial products, service methods, and risk management tools.
